# Mechanism of Sperm Immobilization by *Escherichia coli*

**DOI:** 10.1155/2010/240268

**Published:** 2010-03-30

**Authors:** Vijay Prabha, Ravneet Sandhu, Siftjit Kaur, Kiranjeet Kaur, Abha Sarwal, Ravimohan S. Mavuduru, Shravan Kumar Singh

**Affiliations:** ^1^Department of Microbiology, Panjab University, Chandigarh-160014, India; ^2^Department of Pathology, Government Multi Speciality Hospital, Chandigarh-160016, India; ^3^Department of Urology, Post Graduate Institute of Medical Education and Research, Chandigarh-160012, India

## Abstract

*Aim*. To explore the influence of *Escherichia coli* on the motility of human spermatozoa and its possible mechanism. *Methods*. Highly motile preparations of spermatozoa from normozoospermic patients were coincubated with *Escherichia coli* for 4 hours. At 1, 2 and 4 hours of incubation, sperm motility was determined. The factor responsible for sperm immobilization without agglutination was isolated and purified from filtrates. *Results*. This report confirms the immobilization of spermatozoa by *E. coli* and demonstrates sperm immobilization factor (SIF) excreted by *E. coli*. Further this factor was purified by ammonium sulfate precipitation, gel permeation chromatography, and ion-exchange chromatography. Purified SIF (56 kDa) caused instant immobilization without agglutination of human spermatozoa at 800 *μ*g/mL and death at 2.1 mg/mL. Spermatozoa incubated with SIF revealed multiple and profound alterations involving all superficial structures of spermatozoa as observed by scanning electron microscopy. *Conclusion*. In conclusion, these results have shown immobilization of spermatozoa by *E. coli* and demonstrate a factor (SIF) produced and secreted by *E. coli* which causes variable structural damage as probable morphological correlates of immobilization.

## 1. Introduction

Infections of the male genitourinary tract represent a significant health care problem and account for almost 15% of cases of male infertility [1]. Infections can affect different sites of the male reproductive tract, such as the testis, the epididymis, and male accessory sex glands. Spermatozoa subsequently can be affected by infections at different points in their development and maturation. Acute or chronic infections can compromise spermatogenesis, resulting in quantitative and qualitative reductions [2]. Direct interactions with pathogenic bacteria or immune competent cells represent another possibility for infectious impact on spermatozoa [3, 4]. Genitourinary infections are also associated with biologic and biochemical changes in the seminal plasma that can impair function and fertilizing potential of spermatozoa [5].

Among bacterial species that interact with spermatozoa are well-known causative pathogens of genitourinary infections such as *Escherichia coli, Ureaplasma urealyticum, Mycoplasma hominis,* and *Chlamydia trachomatis* [6]. *E. coli* probably represents the most frequently isolated microorganism in genitourinary infections [7]. It rapidly adheres to human spermatozoa in vitro, resulting in agglutination of spermatozoa. A profound decline in motility of spermatozoa is evident over time caused by severe alterations in sperm morphology [8]. 

The direct inhibitory effect of *E. coli* on progressive motility of spermatozoa is found to depend upon the bacterial concentration. A distinct inhibitory effect is observed with concentration of 10^6^ and agglutination with 10^7^ *E. coli*/mL [9].

Bacterial concentrations utilized in in vitro experiments are much higher than would ever be recoverable from ejaculate specimens. Similar discrepancies between in vitro studies and actual in vivo infections have been observed in tests for the inducibility of the acrosome reaction in artificially and physiologically infected semen samples. None of these phenomena that are evident in vitro have clearly been documented in semen specimens of patients with genitourinary infections [10].

As bacterial concentrations required for affecting sperm motility, morphology and function are rather high, other investigations are being attempted to identify bacterially secreted products that affect the performance of human spermatozoa. Specific bacterial toxins and compounds have not been identified to impair clearly any relevant function of spermatozoa. Therefore, the present work was undertaken to study the mechanism of *E. coli* mediated immobilization of spermatozoa.

## 2. Materials and Methods

### 2.1. Microorganisms

The bacterial isolates used in the present study were isolated from semen samples of infertile males, undergoing semen analysis at Government Multi Speciality Hospital (GMSH), Sector 16, Chandigarh and special infertility clinic at Department of Urology, Post Graduate Institute of Medical Education and Research (PGIMER), Chandigarh, India. 

### 2.2. Isolation of Microorganism from the Ejaculates of Infertile Males

Before taking the semen samples, the patient's recent history was taken into consideration. The semen samples were taken from only those males who did not have antibiotic intake for at least a week. Sperm samples were obtained by masturbation, following a 24-hour continence period, into a sterile wide-mouth beaker. Samples underwent liquefaction at room temperature for 30 minutes. Then, the samples were streaked on blood agar and Mac Conkey plates separately; the plates were incubated aerobically at 37°C for 24–48 hours and observed for the bacterial growth. The isolates obtained were subjected to various tests of identification according to the characteristics laid down in the Bergey's Manual of Determinative Bacteriology [11]. In the present study, 26 isolates were obtained from the 20 semen samples. The various microorganisms included *Pseudomonas aeruginosa*, *Escherichia coli, Micrococcus* sp., *Staphylococcus* sp. and *Bacillus* sp. In total, four isolates of *E. coli* were obtained.

### 2.3. Spermatozoa from Human Males

Semen samples were obtained from healthy male donors (10) by masturbation into sterile wide-mouth containers. The ejaculates were collected from the clinical laboratory of GMSH, Sector 16, Chandigarh and special Infertility clinic at Department of Urology, PGIMER, Chandigarh. Only ejaculates showing normal semen parameters according to WHO criteria [12] were used Samples underwent liquefaction at room temperature for 30 minutes. Experiments were performed within 1 hour of obtaining samples. 

### 2.4. Screening of Isolates for Human Sperm Immobilization

The screening of all the four isolates of *E. coli* obtained from the ejaculates of infertile males (20) for interaction with human sperm identified only one isolate E4 showing sperm immobilization without agglutination (by secreting the factor extracellularly), whereas the other three isolates showed sperm immobilization along with agglutination. Therefore, following experiments focused on the interaction between sperm and *E. coli,* isolate E4.

The isolate was grown in Brain Heart Infusion broth (BHI) under shaking conditions (150 rpm) at 37°C for 72 hours. The culture was centrifuged at 10,000 rpm for 15 minutes at 4°C and cell-free supernatant was prepared by passing the supernatant through a 0.22 *μ*m Millipore filter. Equal volumes of semen sample and cell culture/cell-free supernatant/washed cells were mixed and incubated at 37°C for 30 minutes, 1 hour, 2 hours, and 4 hours, and immobilization of spermatozoa was observed under a light microscope at X400. As control, a sterile growth medium was used. 

### 2.5. Extraction and Purification of Spermatozoal Immobilization Factor (SIF)

72-hour old culture of *E. coli *grown in BHI broth was centrifuged at 10,000 rpm for 15 minutes at 4°C and the supernatant so obtained was subjected to ammonium sulphate precipitation so as to get 20, 40, 60, 80, and 100% saturation. The precipitates so obtained were dissolved in a minimum amount of PBS (50 mM, pH 7.2). The precipitated protein was dialyzed against distilled water under cold conditions and checked for sperm immobilization. Further purification was done by molecular sieving and DEAE-cellulose chromatography. 

#### 2.5.1. Molecular Sieving

Purification of the factor was further done by gel filtration through a Sephadex G-100 (Pharmacia fine chemicals, Uppsala), column (2 cm × 31 cm) equilibrated, and eluted with PBS (50 mM, pH 7.2). Fractions of 3 mL each were collected and each fraction was read at 280 nm on U.V. spectrophotometer. Fractions showing the immobilization of spermatozoa were pooled and concentrated using polyethylene glycol (PEG) 6000 under cold conditions.

#### 2.5.2. DEAE-Cellulose Column Chromatography

The pooled and concentrated fractions after molecular sieving through G-100 were passed through DEAE cellulose, an anion exchange column. First of all, 80 mL of elution buffer (50 mM pH 7.2) were allowed to run down the column. Final elution was done with 0.05, 0.1, 0.2, 0.4, and 0.6 M NaCl dissolved in PBS (50 mM, pH 7.2). Fractions of 4 mL each were collected and read at 280 nm on U.V. spectrophotometer. The fractions causing immobilization of spermatozoa were pooled and concentrated. To verify the purification status of all the preparations, polyacrylamide gel electrophoresis (PAGE) was carried out (10% resolving and 5% stacking gel).

#### 2.5.3. Molecular Weight Estimation

The molecular weight of the protein was estimated by sodium dodecyl sulphate polyacrylamide gel electrophoresis (SDS-PAGE) using the standard molecular weight markers [13]. A 12% gel was prepared in this case and accordingly SDS was added. Rest of the gel preparation was similar to PAGE. After the gel was run, coomassie blue staining was done and molecular weight was estimated.

### 2.6. Effect of SIF on Spermatozoal Motility

Minimum concentration of SIF showing 100% sperm immobilizing activity was determined by mixing different concentrations of SIF (100 *μ*g/mL to 1000 *μ*g/mL) with human spermatozoa (40 × 10^6^ cells/mL). Immediately and after 30 minutes of incubation, the highest dilution of SIF that displayed 100% immobilization of motile spermatozoa was taken as the minimum concentration. Eosin staining [14] was performed to check for viability of spermatozoa on treatment with SIF.

### 2.7. Scanning Electron Microscopy

Processing of samples was done according to the method described by Hafez and Kanagawa [15] with slight modifications. 200 *μ*L of washed sperm suspension (containing 40 × 10^6^ spermatozoa/mL) were incubated with 200 *μ*L of purified SIF for 30 minutes. To each tube, 4 mL of 2.5% phosphate buffered glutaraldehyde were added to the mixture gently with a pasteur pipette. After 30 minutes, samples were centrifuged for 10 minutes at 600 rpm and washed twice in PBS (50 mM, pH 7.2). Control was prepared in the same manner, instead of SIF, PBS was added.

One drop of fixed and washed spermatozoa was placed on a silver painted adhesive tape mounted on brass stubs and air dried. 100 Ǻ gold coating was done on Jeol fine coat ion sputter (JFC-1100). The specimens were observed in Jeol scanning microscope (JSM-6100, Japan) and operated at 20 KV.

## 3. Results

### 3.1. Isolation of Microorganism

The screening of various isolates obtained from the ejaculate of infertile males for the interaction with human spermatozoa identified one isolate of *Escherichia coli* causing immobilization of human spermatozoa by secreting the factor extracellularly. This isolate was selected for further studies.

### 3.2. Extraction and Purification of Spermatozoal Immobilization Factor (SIF)

**Figure 1:**
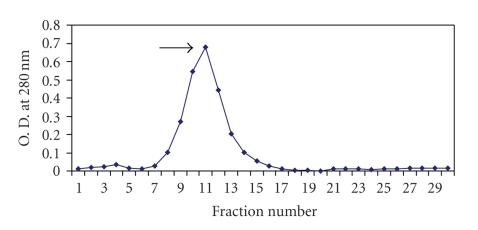
Elution pattern of sperm immobilization factor from *E. coli* on Sephadex G-100 column.

**Figure 2:**
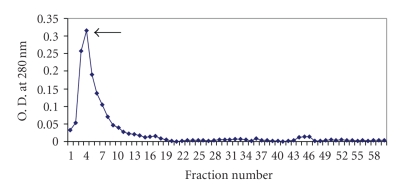
Elution pattern of sperm immobilization factor from *E. coli* on DEAE cellulose column.

Upon ammonium sulphate fractionation, SIF could be precipitated out at 60%–80% saturation. This precipitated fraction was redissolved in phosphate buffer saline (50 mM, pH 7.2) and applied on Sephadex G-100 column. The column chromatographic pattern showed that the immobilization activity was present in the fractions 8–14 with a peak value in fraction 11 where each fraction was of 3 mL quantity (Figure 1). The fractions showing immobilizing activity were pooled and concentrated using polyethylene glycol (PEG 6000) and were again checked for the sperm immobilizing activity. The concentrated fractions from Sephadex G-100 column were applied to DEAE cellulose column. The fractions showing sperm immobilizing activity were 3–7 with peak values in fraction 4 where each fraction was of 4 mL (Figure 2). These fractions were again pooled and concentrated by PEG 6000. The concentrated Ion exchange fractions, when subjected to PAGE, resulted in single protein band.

### 3.3. Molecular Weight Estimation

**Figure 3:**
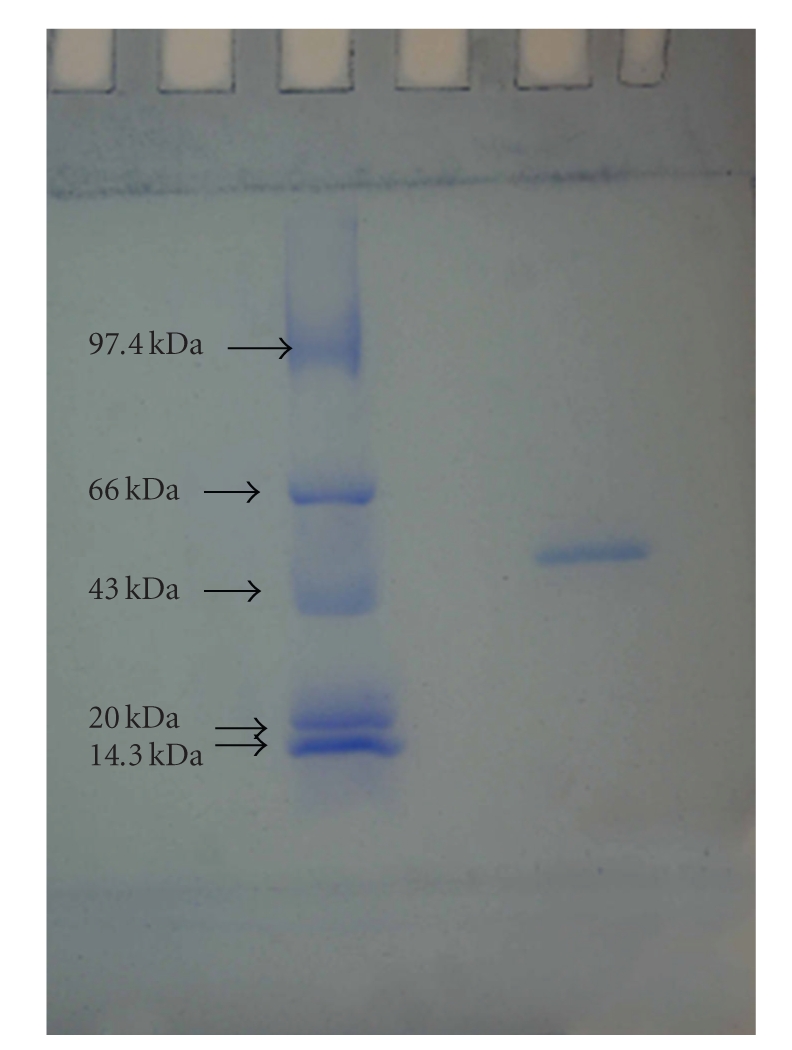
SDS-PAGE showing a purified band of 56 kDa.

The molecular weight of SIF was estimated by sodium dodecyl sulphate polyacrylamide gel electrophoresis (SDS-PAGE). From the results, it could be observed that the purified SIF has molecular weight of ~56 kDa (Figure 3), compared to standard protein markers used.

### 3.4. Minimum Concentration of SIF Showing 100% Immobilization

**Figure 4:**
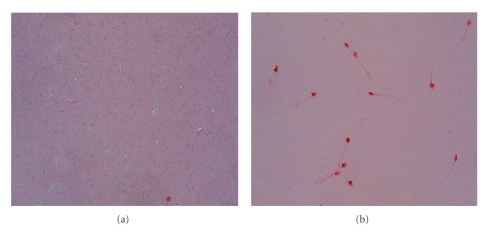
Eosin staining of human spermatozoa after 20s of incubation with (a) PBS, unstained live spermatozoa (b) SIF (2.1 mg/mL), pink stained dead spermatozoa.

When SIF was mixed with human spermatozoa at different concentrations (100–1000 *μ*g/mL), the results showed that complete sperm immobilization could be observed at a minimum concentration of 500 *μ*g/mL after 30 minutes of incubation, while 800 *μ*g/mL of SIF were required to cause complete immobilization of spermatozoa immediately (20s). At a concentration of 2.1 mg/mL, SIF not only caused immobilization of spermatozoa but also had a spermicidal effect (Figures 4(a) and 4(b)). 

The human spermatozoa completely immobilized with 800 *μ*g/mL of SIF when washed with PBS and resuspended in PBS to observe for reversibility of immobilization which showed that immobilization was irreversible.

### 3.5. Scanning Electron Microscopy

**Figure 5:**
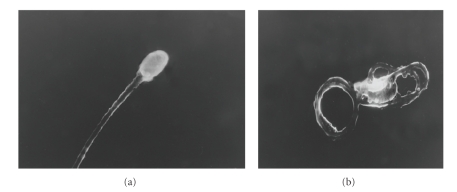
Scanning electron micrograph showing (a) normal washed human spermatozoa (X8, 000 magnification), (b) human spermatozoa on treatment with SIF showing curling of tail and morphological changes (X6, 000 magnification).

Scanning electron microscopy of washed human spermatozoa treated with purified SIF showed multiple and profound alterations in the human spermatozoa (Figures 5(a) and 5(b)). Morphological alterations involved all the superficial structures of spermatozoa such as head, mid piece, neck, and tail.

## 4. Discussion

Male urogenital tract infection is an important cause of male infertility. The etiological role of infections in male infertility has been paid attention in recent years. Infectious processes may lead to deterioration of spermatogenesis, impairment of sperm function and obstruction of the seminal tract [7]. As a result, microbiological investigation can reveal a probable infection. 35.22% infertile men are positive at least for one pathogen and there is an important relation between the bacteriospermia and sperm impairment [16]. 

*Escherichia coli* is one of the main microorganism isolated from the semen with the most negative influence on sperm motility and morphology. Other common microorganisms generally isolated from semen are *Staphylococcus aureus, gonococci, Candida* sp., and *Klebsiella* sp. [17].

Although several research groups have outlined the negative influence of various *E. coli* strains on motility and motility parameters of human spermatozoa in in vitro experiments but the mechanism of this immobilization has still not been elucidated [18, 19]. Some authors have suggested that direct interactions between bacteria and human spermatozoa represent the bacterial mechanism that facilitates immobilization of spermatozoa. Direct interactions of bacteria and spermatozoa have been discovered for different bacterial species such as *E. coli* [9], Mycoplasmas, *U. urealyticum* [20], and *Chlamydia* species [21]. Immobilization of spermatozoa is associated with tight adhesins between bacteria and spermatozoa resulting in agglutination of spermatozoa. Adhesion and agglutination is followed by profound alterations and damage of the ultra structure of spermatozoa as viewed by scanning and transmission electron microscopy. Other investigators have reported evidence for soluble spermatotoxic factors with low molecular weight produced and secreted by pathogenic bacteria [22]. This study also presents immobilization of spermatozoa by *E. coli* obtained from the ejaculate of males attending infertility clinic. Immobilization of spermatozoa seemed to be associated with the factor released into the extracellular medium as when the washed cells, cell-free supernatant, and cell culture were checked for sperm immobilization potential after 72 hours of growth, no immobilization occurred with the washed cells. 

Further, an attempt was made to isolate and purify sperm immobilization factor from *E. coli* culture supernatant. When the culture supernatant was subjected to ammonium sulphate precipitation, most of the SIF was precipitated at 60%–80% saturation.

The precipitated factor redissolved in PBS (50 mM, pH 7.2) was further purified by filtration through a Sephadex G-100 column. The column chromatographic pattern showed that SIF was present in fractions 8–14. These fractions were pooled, concentrated and applied to DEAE cellulose column. The results indicated that most of the SIF could be eluted with PBS. The fractions showing immobilization (3–7) were pooled and concentrated and subjected to PAGE, showing one major protein band.

The purified factor not only had a remarkable sperm immobilizing activity, but also it had a spermicidal effect. Spermatozoa immobilized by SIF after removing the SIF and resuspended in buffer did not gain motility indicating that this phenomenon is irreversible.

Earlier, Paulson and Polakoski [22] also isolated sperm immobilization factor from *E. coli* cultures. The factor was stable to heating, freezing, and lyophilization and immobilized but did not kill spermatozoa. The factor passed through the dialysis tubing and through a 500 molecular weight cut off membrane, indicating that it is a relatively small component. Further, spermatozoa immobilized by SIF isolated from *E. coli* were rendered motile by removing the factor and resuspending them in normal seminal plasma indicating that the mechanism is reversible.

However, the factor isolated in the present studies is heat labile. It not only immobilized the spermatozoa but at higher concentration also had a spermicidal effect. Moreover, the factor was nondialyzable and spermatozoa once immobilized, after centrifugation and resuspension in buffer, did not revert to motility indicating the mechanism to be irreversible.

Electron microscopic analysis of spermatozoa-SIF interactions revealed multiple and profound alterations in the structure of spermatozoa. Morphological alterations involved all superficial structures of spermatozoa in particular curling of the tail indicating that morphological defects might be accounting for the immobilization of spermatozoa by *E. coli*. These results are in accordance to earlier study made by Diemer et al. [8] wherein they have reported the negative influence of *E. coli* on the motility of spermatozoa as a consequence of *E. coli*-induced ultra structural alterations.

Although, the nature of the SIF causing immobilization of spermatozoa is not known yet, but the results obtained from this study do indicate that the bacteria causing genital tract infection could have a deficient role in the motility of men's spermatozoa.
